# Manufacture of titanium alloy materials with bioactive sandblasted surfaces and evaluation of osseointegration properties

**DOI:** 10.3389/fbioe.2023.1251947

**Published:** 2023-08-21

**Authors:** Jie Wang, Baohui Yang, Shuai Guo, Sen Yu, Haopeng Li

**Affiliations:** ^1^ Department of Orthopedic Surgery, Second Affiliated Hospital of Xi’an Jiaotong University, Xi’an, China; ^2^ Shaanxi Key Laboratory of Biomedical Metal Materials, Northwest Institute for Nonferrous Metal Research, Xi’an, China

**Keywords:** biomaterial, titanium alloy, surface modification, bioactive sandblasted surface, osseointegration

## Abstract

Titanium alloys are some of the most important orthopedic implant materials currently available. However, their lack of bioactivity and osteoinductivity limits their osseointegration properties, resulting in suboptimal osseointegration between titanium alloy materials and bone interfaces. In this study, we used a novel sandblasting surface modification process to manufacture titanium alloy materials with bioactive sandblasted surfaces and systematically characterized their surface morphology and physicochemical properties. We also analyzed and evaluated the osseointegration between titanium alloy materials with bioactive sandblasted surfaces and bone interfaces by *in vitro* experiments with co-culture of osteoblasts and *in vivo* experiments with a rabbit model. In our *in vitro* experiments, the proliferation, differentiation, and mineralization of the osteoblasts on the surfaces of the materials with bioactive sandblasted surfaces were better than those in the control group. In addition, our *in vivo* experiments showed that the titanium alloy materials with bioactive sandblasted surfaces were able to promote the growth of trabecular bone on their surfaces compared to controls. These results indicate that the novel titanium alloy material with bioactive sandblasted surface has satisfactory bioactivity and osteoinductivity and exhibit good osseointegration properties, resulting in improved osseointegration between the material and bone interface. This work lays a foundation for subsequent clinical application research into titanium alloy materials with bioactive sandblasted surfaces.

## 1 Introduction

Titanium and titanium alloys have become popular materials in the field of orthopedic implants because of their good mechanical properties, corrosion resistance and biocompatibility (Pobloth et al., 2018; Kaur and Singh, 2019; Li et al., 2020). The most widely used titanium alloy material in clinic is Ti6Al4V. However, with the wide application of Ti6Al4V in orthopedics, some shortcomings of Ti6Al4V have gradually emerged that have led to implant failure. For example, Ti6Al4V does not exhibit bioactivity and osteoinductivity, and releases ions on the surfaces of materials in which it is used (Panayotov et al., 2015; Xu et al., 2019; Hsu et al., 2020). Additionally, its lack of bioactivity and osteoinductivity is considered to be the main cause of implant loosening and revision surgery (Li et al., 2014; Mistry et al., 2016). Furthermore, the uptake of excess metal ions by cells can affect DNA replication and even lead to cell death; aluminum (Al) and vanadium (V) ions on the surface of Ti6Al4V have some potential toxic effects on human body. V ions can cause detrimental tissue reactions and cytotoxicity, and there is evidence that Al ions are involved in the mechanism of long-term Alzheimer’s disease (Bedi et al., 2009; Alshammari et al., 2022). Due to the above issues, limiting Ti6Al4V’s osseointegration properties, thus the osseointegration between titanium alloy materials and bone interfaces is simply unsatisfactory and can lead to total implant failure.

Good osseointegration, which refers to the direct structural and functional connection between bone and material surface, is considered to be the most important prerequisite of successful implantation (Kim et al., 2021). Good osseointegration properties of the implant material are necessary factors for achieving osseointegration, such as bioactivity and osteoinductivity. Hence, the ideal orthopedic implant material should have good osseointegration properties. However, Ti6Al4V does not meet this critical requirement by itself. Therefore, there is still a profound need to develop novel implant materials capable of good osseointegration properties.

Surface modification can be used to change the surface morphology of materials and only changes the surface properties of the material but also preserves its internal properties (Bai et al., 2018; Zhou et al., 2018; Geng et al., 2021). Because it changes the surface morphology and elemental composition of materials to some extent, surface modification is considered to be an effective method to improve the osseointegration properties of the material. In order to improve the osseointegration properties of implant materials, various surface modification techniques have been applied since the osseointegration properties of implant materials are closely related to surface morphology (surface morphological characteristics, surface roughness) and physicochemical properties (elemental composition, surface wettability) of the material (Ehlert et al., 2021; Geng et al., 2022).

In this study, we proposed a novel surface modification method using specific particles, some of which remained on the surface of substrate material after a sandblasting process. We refer to our method as a novel sandblasting surface modification process. Compared to the smooth Ti6Al4V titanium alloy commonly used in clinic, the titanium alloys treated by the novel sandblasting surface modification process not only changes the surface morphology characteristics of the material, but also changes the chemical composition ratio of the surface of the material. This may greatly affect the osseointegration properties of the material and thus the osseointegration between titanium alloy materials and bone interface, and was therefore the central focus of this study. In this study, the experimental group was the titanium alloy with bioactive sandblasted surface, while the control group was the smooth Ti6Al4V titanium alloy commonly used in clinic.

## 2 Materials and methods

### 2.1 Manufacture of the titanium alloy with bioactive sandblasted surfaces

The Ti6Al4V specimens treated by novel sandblasting surface modification process were labeled as the sandblasted titanium alloy group, and the Ti6Al4V specimens with just surface polishing were labeled as the smooth titanium alloy group.

#### 2.1.1 Sandblasted titanium alloy group

To begin our novel sandblasting process, we first degreased Ti6Al4V round specimen (diameter 10 mm, thickness 2 mm, for the *in vitro* experiment) (Northwest Institute for Non-ferrous Metal Research, China) and Ti6Al4V square specimen (length 15 mm, width 10 mm, thickness 2 mm, for the *in vivo* experiment) (Northwest Institute for Non-ferrous Metal Research, China) with acetone. Each specimen surface was then treated with 2% nitric acid, 2% hydrofluoric acid, and 2% ammonium fluoride at 55°C for 30 s and then ultrasonically cleaned with deionized water. After this, the specimens were surface treated with 50 μm diameter mixed sand materials (containing silicon dioxide and a small amount of Mg and Fe) (Northwest Institute for Non-ferrous Metal Research, China) at a pressure of 0.8 MPa and a distance of 3 cm from the surface. The injection direction was an 80° angle to the surface, and the sandblasting treatment time was 60 s. In addition, after ultrasonic cleaning using distilled water for 15 min, we dried each specimen at 50°C for later use. Finally, three screw holes with diameters of 1 mm were drilled in the square specimen. The samples were sterilized by autoclaving prior to cell culture and animal experiments.

#### 2.1.2 Smooth titanium alloy group

For the smooth-polished specimens for the control group, we once again first degreased Ti6Al4V round specimen (diameter 10 mm, thickness 2 mm, for the *in vitro* experiment) (Northwest Institute for Non-ferrous Metal Research, China) and Ti6Al4V square specimen (length 15 mm, width 10 mm, thickness 2 mm, for the *in vivo* experiment) (Northwest Institute for Non-ferrous Metal Research, China) with acetone and then each specimen surface was treated with 2% nitric acid, 2% hydrofluoric acid, and 2% ammonium fluoride at 55°C for 30 s and cleaned them with deionized water. Next, the specimens underwent 600 mesh and then 1000 mesh sandpaper grinding and polishing. In addition, after ultrasonic cleaning using distilled water for 15 min, we dried each specimen at 50°C for later use. Finally, three screw holes with diameters of 1 mm were drilled in the square specimen. The samples were sterilized by autoclaving prior to cell culture and animal experiments.

### 2.2 Characterization of the specimen surface

#### 2.2.1 Surface morphology

We used scanning electron microscopy (SEM) to observe and analyze the surface morphology of the smooth titanium alloy and sandblasted titanium alloy groups under the condition of 20Kv 500 imes.

#### 2.2.2 Surface chemical composition

To analyze the chemical compositions of the specimens, we randomly selected three different scanning areas on the surface of the two groups of specimens. We then observed and characterized the types and contents of chemical elements on the surface of the two groups using an energy dispersion spectrometer (EDS, NCA X-Act, Sirion, America) with acceleration voltage set to 15 kV. The atomic force microscope (AFM) (Dimension Icon, Bruker, America) was used to analyze the average roughness (Ra) and root-mean-square roughness (Rq) of samples in smooth titanium alloy group and sandblasted titanium alloy group, with the scanning range of 15 μm × 15 μm. In addition, X-ray photoelectron spectroscopy (XPS, ESCALAB 250Xi, Thermo Scientific, America) with an Al K*α* radiation source was used to analyze the elemental states on the surface of the two groups of specimens, and we used a multifunctional X-ray diffractometer (XRD, D8 ADVANCE, Bruker, Germany) with Cu K*α* radiation between 2*θ* of 20° and 90° to analyze the phase of the two groups of specimens as well.

#### 2.2.3 Surface wettability

The surface wettability of the two groups was assessed by the contact angle measurements (DO4222Mk1, Kruss, Germany) at ambient temperature and humidity. To take our measurements, we put 3 μL of deionized water drops on the surface of the specimen and let it stand for 5 s before detection. Water contact angle measurements were thusly obtained by repeated measurements of randomly selected areas of each specimen.

### 2.3 *In vitro* experimentation

#### 2.3.1 Cell culture and identification

For our *in vitro* experiments, the ilium bone of a 2-month-old New Zealand white rabbit was washed with PBS (including double antibodies) 3 times and divided into bone blocks of about 1 × 1 × 1 mm^3^ in size. After washing with PBS, 0.1% type I, Ⅱ, and Ⅳ collagenase and dispase were added, and left to digest overnight at 4°C. After filtration of the above mixture, the filtrate was centrifuged at 1,000 rpm/min for 5min, the supernatant was discarded, and the precipitation was completely suspended on the culture medium. The precipitation was evenly spread in the culture flask, and the osteoblast medium (containing 10% fetal bovine serum, 1% double antibody, and 1% osteogenic growth factor) was then added. Next, we placed the culture in a 5% CO2 incubator with a constant 37°C temperature and changed the liquid every 48 h. In addition, the osteoblasts were purified by the differential adhesion method and then got the third generation osteoblasts for use. We identified osteoblasts using a modified calcium-cobalt method, and then seeded the osteoblasts that were cultured to the third generation on 6-well plate cells with a concentration of 5×10^4^/mL. After 48 h of culture, the cell plates were fixed with 95% ethanol for 10 min and then washed with distilled water. We then immersed the cells in incubation solution (12 mL 2% barbiturate sodium, 3 mL 2% magnesium sulfate, 12 mL 3%β -sodium glycerophosphate, 20 mL 2% calcium chloride, and 22 mL distilled water), bathed them in water at 37°C for 4 h, washed them with distilled water, and then treated them with 2% cobalt nitrate solution for 4 min, fully washing them with distilled water to eliminate excess cobalt nitrate. The cells were then immersed in 1% ammonium sulfide solution for 3 min and washed with distilled water and sealed for observation after air drying.

#### 2.3.2 Cell proliferation

The proliferation of osteoblasts on the specimens was detected using a cell counting assay kit-8 (CCK-8). To accomplish this, we co-cultured the two groups of specimens separately with the third generation osteoblasts on 24-well plates. First, 1 mL osteoblast suspension with a density of 5×10^4^ was inoculated on the surface of the two groups of specimens placed on the 24-well plate and co-cultured for 1, 4, and 7 days. At the time of culture, the medium was removed, and the samples were washed twice with PBS and transferred to a new 24-well plate. We then added 500 µL of osteogenic medium and CCK-8 reagent (10:1 by volume) to each well and culture at 37°C for 4 h at 5% CO2 concentration. We then measured the absorbance values at 450 nm by spectrophotometry.

#### 2.3.3 Detection of alkaline phosphatase (ALP) activity

We detected the ALP activity of the osteoblasts on the surface of the two groups of specimens by ALP detection kit (Sigma, USA). Here, the third generation osteoblasts were separately co-cultured with the two groups of specimens on 24-well plates, and 1 mL osteoblast suspension with a density of 5×10^4^ was inoculated on the surface of the two groups of specimens for 7 days and 14 days, respectively. Cell suspension was then also prepared at 7 and 14 days of culture, respectively. Then, following the instructions of the ALP detection kit (Sigma, United States), we added 960 μL reaction buffer to the two groups of wells of the 96-well plate, and also added 20 μL pNPP solution to the two groups of wells. The mixture was then allowed to stand at 37°C for 10 min until it was evenly mixed. Next, we added 20 μL of the corresponding co-cultured osteoblast suspension to the two groups of wells and immediately detected the absorbance value at 405 nm and 30 min. We repeated this absorbance value measurement 3 times and took the average of the values so that the measured absorbance value could indirectly reflect the ALP activity of osteoblasts.

#### 2.3.4 Detection of osteogenesis-related gene expression

Quantitative real-time reverse-transcriptase polymerase chain reaction (qRT-PCR) was used to analyze the expression of osteogenesis-related genes on the surface of the two groups of specimens quantitatively. Each specimen was first inoculated with 1 mL of cell suspensions at a density of 5×10^4^ cells per well and co-cultured for 7 and 14 days. We extracted total RNA with TRIZOL reagent (Invitrogen) on days 7 and 14 and detected the purity of RNA by UV-vis spectrophotometer. We then reverse transcribed the complementary DNA (cDNA) from 1 μg of total RNA using a T100 Thermal Cycler (BIO-RAD, USA) with BeyoRT Ⅱ cDNA reagent containing gDNA Eraser (Beyotime Biotechnology, China). The primer sequences of the selected osteogenesis-related genes and the house-keeping gene are shown in Table 1. Finally, the expressions of the osteogenesis-related genes, including ALP, Runt-associated transcription factor 2 (RUNX2), type I collagen α1 (Col1α1), and osteocalcin (OCN), were analyzed using glyceraldehyde-3-phosphate dehydrogenase (GAPDH) as the house-keeping gene for normalization and quantified by qRT-PCR according to their Ct (cycle threshold) values.

**TABLE 1:** The primer sequences of the selected osteogenesis-related genes and the house-keeping gene.

Gene	Forward primer	Reverse primer
ALP	5′-cgt​gtt​cac​ctt​tgg​agg​at-3′	5′-ctg​ggc​ctg​gta​gtt​gtt​gt-3′
RUNX2	5′-ccc​caa​gta​gcc​acc​tat​ca-3′	5′-gag​gcg​gtc​aga​gaa​caa​ac-3′
Col1α1	5′-cat​caa​ggt​ctt​ctg​cga​ca-3′	5′-ctt​ggg​gtt​ctt​gct​gat​gt-3′
OCN	5′-gtg​cag​agt​ctg​gca​gag​g-3′	5′-ggt​tga​gct​cgc​aca​cct-3′
GAPDH	5′-atc​act​gcc​acc​cag​aag​ac-3′	5′-gtg​agt​ttc​ccg​ttc​agc​tc-3′

#### 2.3.5 Detection of type I collagen secretion

To detect the type I collagen secretion of osteoblasts on the surface of the two groups of specimens, we used immunocytochemical staining. First, the third generation osteoblasts were separately co-cultured with the two groups of specimens on 24-well plates, and 1 mL osteoblast suspension with a density of 5×10^4^ was inoculated on the surface of the two groups of specimens for 7 and 14 days, respectively. After 7 and 14 days of culture, the osteoblasts were prepared as cell suspension and transplanted to cell climbing slices, and the culture was continued for 12 h. Then, we rinsed the cell climbing slices with PBS two to three times and fixed overnight with 4% paraformaldehyde solution. After the 4% paraformaldehyde solution was removed, the cell climbing slices were washed with PBS 3 more times. We then dried the cell climbing slices and used a pen to draw circles on the cover glass where the cells were evenly distributed. These circle-identified areas were then treated with PBS solution containing 10% goat serum and 0.3% Triton X-100 cell lysis solution for 2 h. Next, we added the primary antibody (anti-type I collagen antibody, dilution 1:400) to the samples and incubated them at 4°C overnight. The secondary antibody (Alexa Fluor 488 conjugated goat anti-rabbit IgG, dilution 1:500) was then added to the samples and incubated at 4°C for 50 min. After this, we washed the cell climbing slices 3 times for 5 min each time and let them dry. When the cell climbing slices were dry, we dropped DAPI dye solution into the circle-identified areas and incubated the slices for 10 min at room temperature and away from light. After that, we once again washed the cell climbing slices 3 times for 5 min each time and then dried and sealed the cell climbing slices. Finally, the cell climbing slices were observed under fluorescence microscope, and the images were collected.

#### 2.3.6 Observation of cell growth on the specimen surfaces

We observed the growth and proliferation of osteoblasts on the surfaces of the two groups of specimens by SEM. As mentioned above, the third generation osteoblasts were separately co-cultured with the two groups of specimens on 6-well plates, and 1 mL osteoblast suspension with a density of 5×10^4^ was inoculated on the surface of the two groups of specimens for 7 and 14 days, respectively. After 7 and 14 days of culture, the cells were fixed with a fixing solution of 2.5% glutaraldehyde and 4% paraformaldehyde for 4 h. After that, the specimens were rinsed 4 times with PBS for 20 min each time. Then, the specimens were dehydrated using a gradient series of ethanol solutions (30%, 50%, 70%, 90%, and 100%). Next, the specimens were immersed in isoamyl acetate solution for 15 min and a carbon dioxide critical point dryer was used for drying. After drying, we glued the specimens to the specimen table and then coated them by the sputter process with a metal film 50–300 Å thick. Finally, the specimens were placed under the SEM for observation.

### 2.4 *In vivo* experimentation

#### 2.4.1 Preparation of the animal model

Our animal model preparation experiment was carried out in strict accordance with the relevant regulations of animal protection set forth in the Declaration of Helsinki and was approved by the Biomedical Ethics Committee of the Xi’an Jiaotong University Health Science Center (No. XJTULAC 2019-933). Twenty New Zealand white rabbits (2 months old; male; 2–2.5 kg) were used for the experiment, and these animals were purchased from the Animal Experiment Center of Xi ‘an Jiaotong University Medical College. The rabbits were randomly divided into the smooth titanium alloy group and the sandblasted titanium alloy group according to the random number table method, with 10 rabbits in each group.

The implantation site was the ilium of each rabbit. Firstly, pentobarbital sodium 30 mg/kg was used for intravenous anesthesia. After this, the rabbits underwent skin preparation, and the surgical area was disinfected. The skin and subcutaneous tissues were then incised, and the muscles were bluntly separated in order to expose the ilium fully. After grinding with a grinding drill, we formed a bone groove without bone cortex measuring about 15 × 10 × 2 mm^3^. Next, each rabbit was implanted according to its group. We then rinsed treatment area with normal saline and successively sutured all twenty incisions. After the operation, the rabbits were given subcutaneous injection of antibiotics to prevent surgical site infection, and postoperative feeding time was 8 weeks and 12 weeks. Euthanasia was performed using pentobarbital sodium 200 mg/kg intravenously at each of the two time points mentioned above.

#### 2.4.2 Animal tissue toxicity assay

We used hematoxylin and eosin (HE) staining to detect the toxicity of titanium alloy implants in animal tissues. Twelve weeks after surgery, we collected the adjacent soft tissue of the implants, liver, spleen, and mesenteric lymph node tissues from the two groups of animal models. The tissues were then fixed in 4% paraformaldehyde for 72 h and dehydrated in a gradient of 20%–90% alcohol followed by transparent treatment with xylene. Then, an embedded wax block was fixed on the slicer and sliced with a thickness of 6 μm before drying. Sections were dewaxed by xylene and then dehydrated once again by 20%–90% alcohol gradient. The tablets were then dyed with HE in turn, dehydrated by 20%–90% alcohol gradient yet again, transparently treated with xylene, and sealed with neutral gum. We then examined the processed tissue sections under microscope and collected the images for analysis.

#### 2.4.3 Micro-CT detection of osseointegration

At 8 and 12 weeks postoperatively, iliac bone specimens were collected from both groups of animal models. The removed iliac bone specimens were then immersed in 4% paraformaldehyde solution and fixed for 48h, and we then performed micro-CT scanning on these specimens. We analyzed the scanned data files by micro-CT (YXLON International GmbH, Germany) and analyzed the volume and density of the trabecular bone within 1 mm around the implant surface in particular. The newly-formed bone volume fraction (newly-formed bone volume/total volume×100%) within 1 mm of the implant surface was then calculated by micro-CT (YXLON International GmbH, Germany).

#### 2.4.4 Hard tissue section detection of osseointegration

For detection of osseointegration, the iliac bone specimens were immersed in 4% paraformaldehyde solution and fixed for 48 h and afterward removed and rinsed for 2 h. Next, the specimens were immersed in gradient alcohol solution for dehydration and then embedded in methyl methacrylate. These prepared embedded tissue blocks were then sectioned, and we used Leica SP1600 microtome (Leica, Germany) to slice each target area. We used P400, P800, and P1200 sandpaper to grind and polish the slices before staining them with Van Gieson stain and observing them under optical microscope and photographing them. Our quantitative analysis of osteogenesis on each implant surface began with the random selection of 6 fields (approximately 12 mm^2^) on each implant surface to ensure that the selected areas of the two groups were relatively consistent in size. Finally, we used image analysis software Image-Pro Plus 6.0 to calculate the total area of trabecular bone in each field and conduct statistical analysis.

### 2.5 Statistical analysis

To carry out our statistical analysis, we transformed the measurement data obtained above into the form of mean ± standard deviation and used SPSS 25.0 software to conduct an independent-sample *t*-test for comparison between groups. We considered *p* < 0.05 to indicate a statistically significant test result.

## 3 Results

### 3.1 Specimen presentation

**FIGURE 1:**
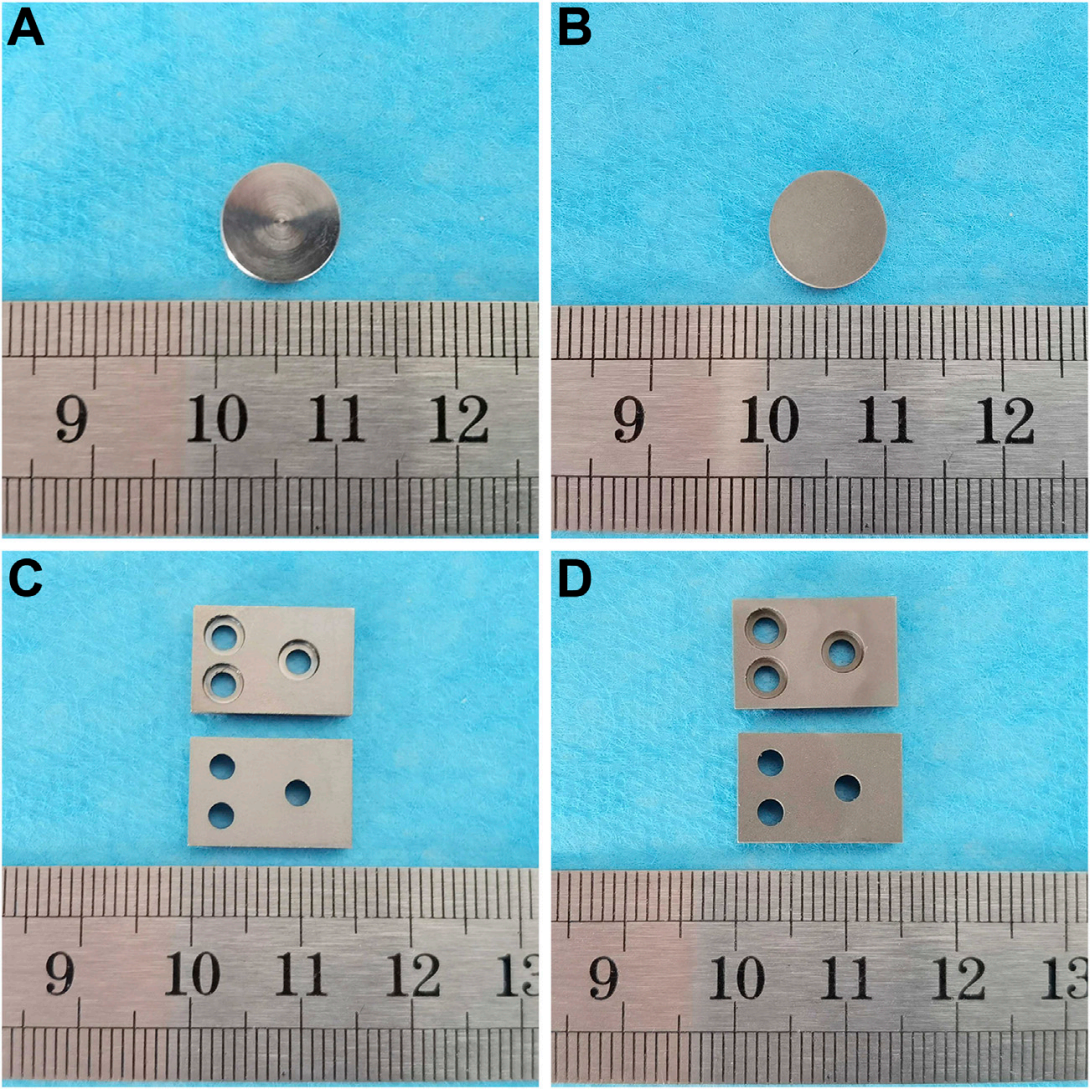
*In vitro* experiment specimens from the **(A)** smooth titanium alloy group and **(B)** sandblasted titanium alloy group. *In vivo* experiment specimens from the **(C)** smooth titanium alloy group and **(D)** sandblasted titanium alloy group.

*In vitro* experiment specimens from the smooth titanium alloy group and sandblasted titanium alloy group are shown in Figures 1A, B. *In vivo* experiment specimens from the smooth titanium alloy group and sandblasted titanium alloy group are shown in Figures 1C, D.

### 3.2 Surface characterization

**FIGURE 2:**
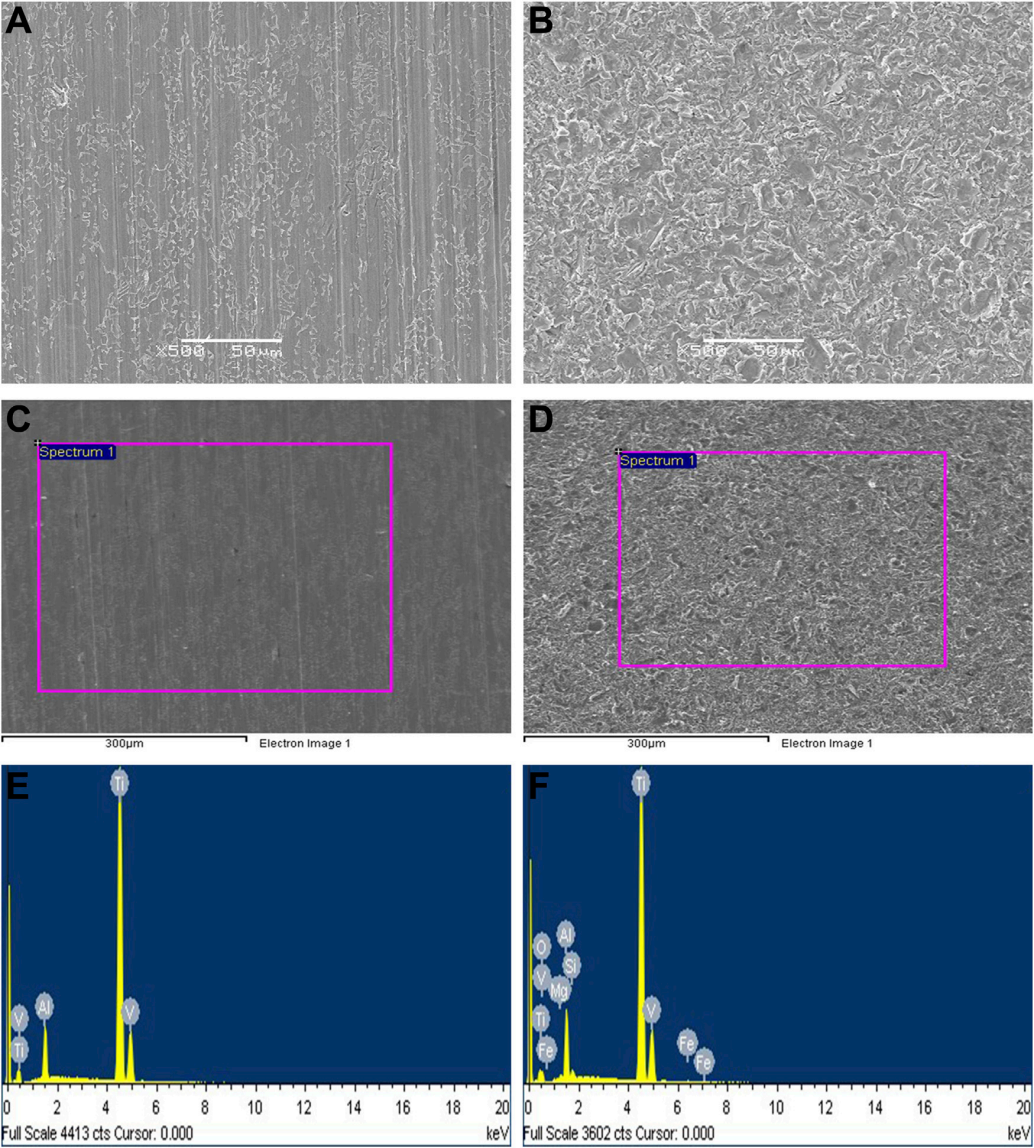
The surfaces of the specimens from the **(A)** smooth titanium alloy group and **(B)** sandblasted titanium alloy group under SEM. EDS detection results. **(C)** detection area of smooth titanium alloy group; **(D)** detection area of sandblasted titanium alloy group; **(E)** EDS detection results of smooth titanium alloy group; **(F)** EDS detection results of sandblasted titanium alloy group.

The surfaces of the specimens from the smooth titanium alloy under SEM is shown in Figure 2A., and the surfaces of sandblasted titanium alloy specimens under SEM is shown in Figure 2B. On the surfaces of the smooth titanium alloy group, we can see scratches in the same direction and punctate cracks and protrusions. However, the bioactive sandblasted surfaces of the sandblasted titanium alloy group show dense and irregularly distributed stacked tile-like bumps. Compared to the smooth titanium alloy group, the surface morphology of the bioactive sandblasted surfaces from the sandblasted titanium alloy group is more diverse.

Our EDS detection results are shown in Figure 2, Table 2, and Table 3. The surface of the specimens from the smooth titanium alloy group contained Ti, Al, and V, and the proportions of the three elements were about 87.77% Ti, 9.12% Al, and 3.10% V (Figures 2C, E; Table 2). From Figures 2D, F, and Table 3, we can see that the bioactive sandblasted surfaces from the sandblasted titanium alloy group were mainly composed of Ti, Al, V, and O and contained trace elements of Mg, Si, and Fe as well. The proportions of each element were 70.61% Ti, 9.17% Al, 2.57% V, 14.71% O, 0.97% Mg, 1.01% Si, and 0.97% Fe. This indicates that new elements were introduced into the bioactive sandblasted surface, changing the chemical composition of the titanium alloy’s surface.

**TABLE 2:** Proportions of elements in surface of the specimens from the smooth titanium alloy group.

Element	Proportion (%)
Al	9.12
Ti	87.77
V	3.10
Total	100.00

**TABLE 3:** Proportions of elements in surface of the specimens from the sandblasted titanium alloy group.

Element	Proportion (%)
O	14.71
Mg	0.97
Al	9.17
Si	1.01
Ti	70.61
V	2.57
Fe	0.97
Total	100.00

**FIGURE 3:**
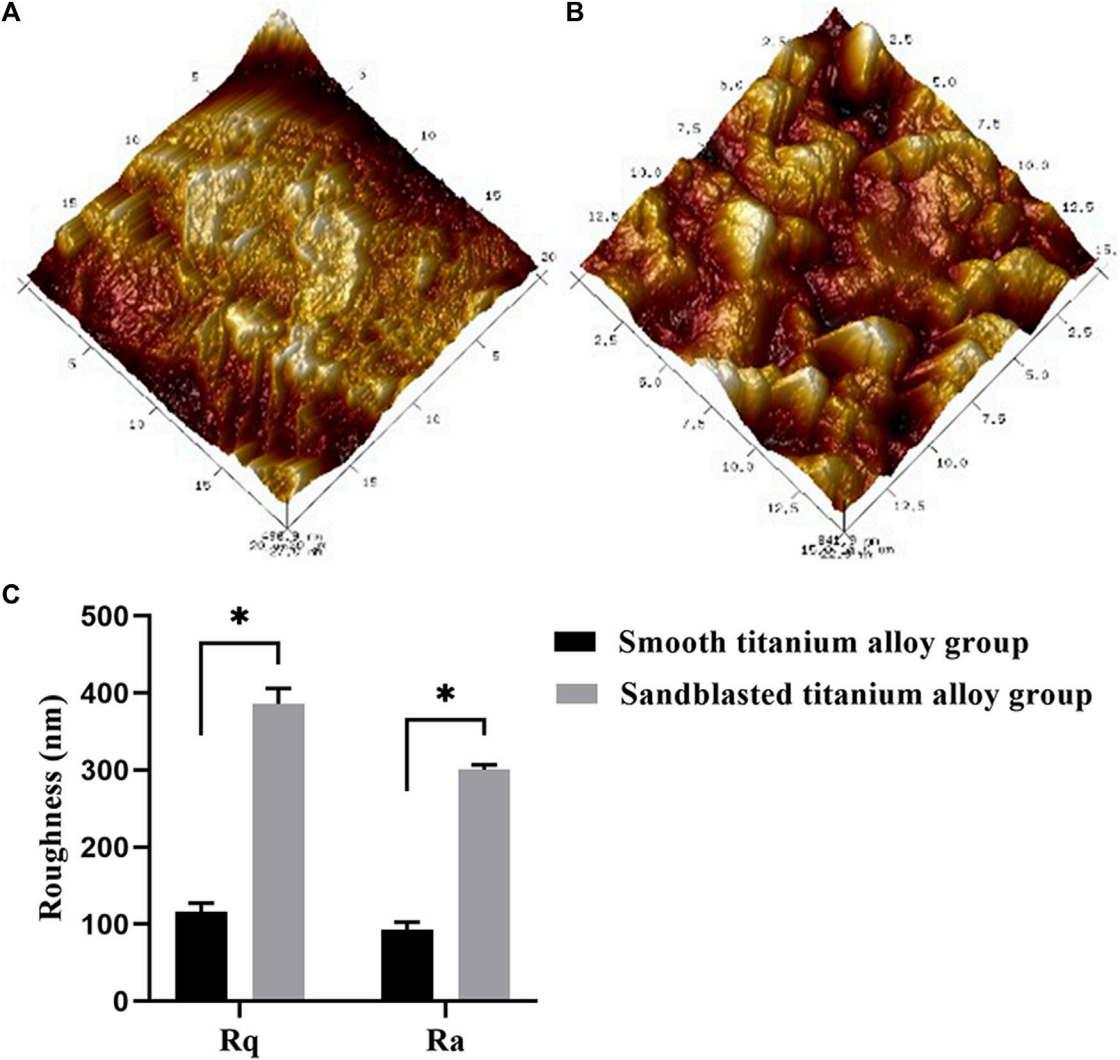
AFM topographical images of **(A)** smooth titanium alloy group and **(B)** sandblasted titanium alloy group, with the range of 15 μm × 15 μm. **(C)** Roughness values (Rq and Ra) of smooth titanium alloy group and sandblasted titanium alloy group, with the range of 15 μm × 15 μm ^*^ *p* < 0.05.

The image of the AFM inspection results and the roughness values are shown in Figure 3. The surfaces of the samples in the smooth titanium alloy group and the sandblasted titanium alloy group exhibited a uniform surface structure. The surface of the samples in the smooth titanium alloy group was basically flat, but there were still scratches remaining (Figure 3A), with Rq and Ra values of 116.23 ± 10.89 and 92.7 ± 10.10 nm (Figure 3C), respectively. Regular groove-like depressions and bumps were present on the surface of the samples in the sandblasted titanium alloy group (Figure 3B), with Rq and Ra values of 386.00 ± 20.14 and 300.50 ± 6.18 nm (Figure 3C), respectively. The novel sandblasting surface modification process increased the roughness of the material surface.

**FIGURE 4:**
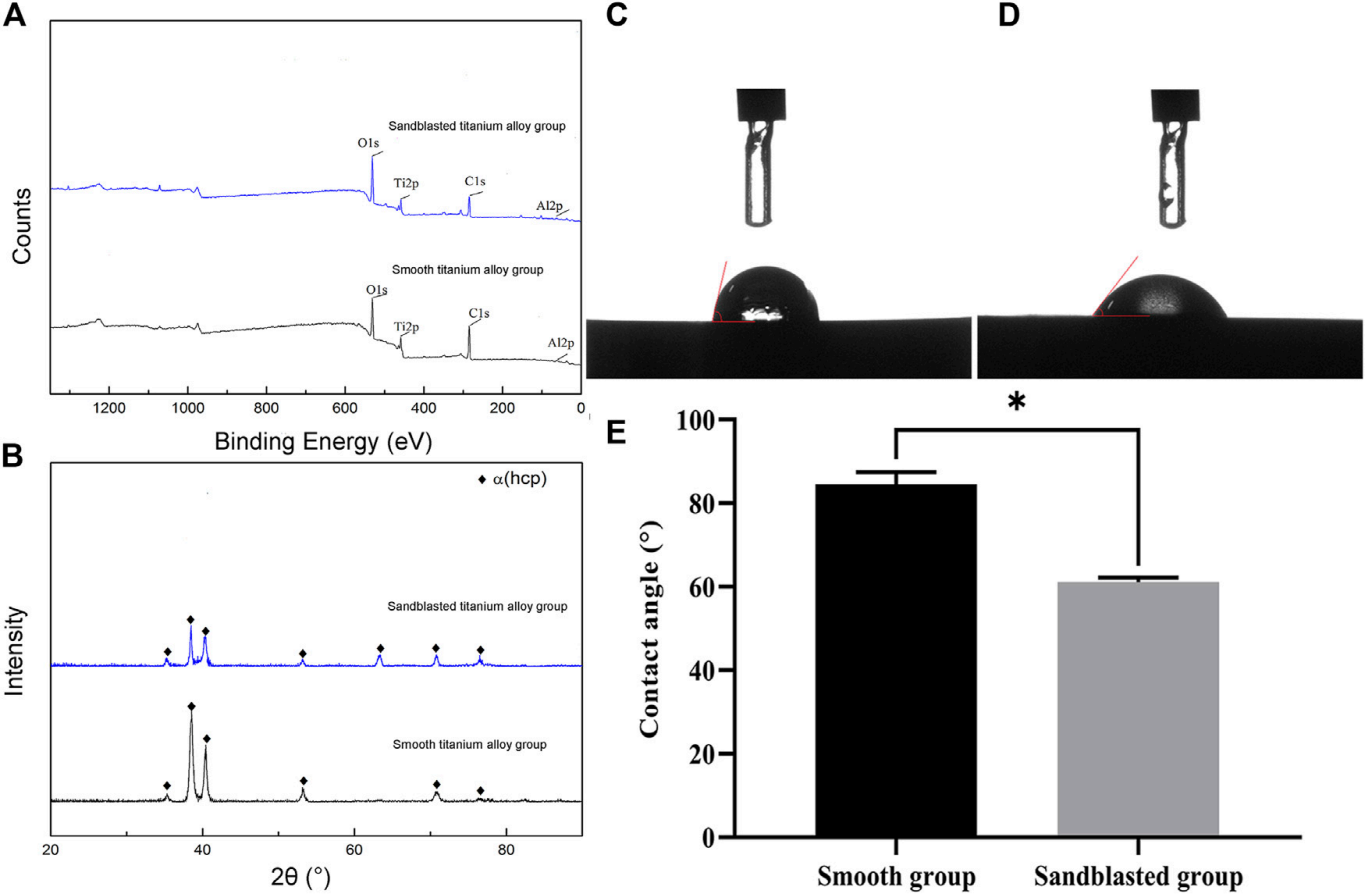
Analysis results of **(A)** XPS and **(B)** XRD. The detection results of contact angle from **(C)** the smooth titanium alloy group and **(D)** sandblasted titanium alloy group. **(E)** Analysis of contact angle between smooth group and sandblasted group. ^*^ *p* < 0.05.

The results of our XPS detection are shown in Figure 4A. Here we see that the bioactive sandblasted surface contained a small amount of Fe and Si because the sandblasting material contained Fe and Si. The results from our XRD analysis are shown in Figure 4B. From this figure we can see that the surfaces of the specimens from both groups were composed of α-Ti phase. Specifically, we see 2θ angles of 35°, 38°, 40°, 53°, 62°, 70°, and 76° diffraction peaks corresponding to α-Ti phase, and (100), (002), (101), (102), (2-10), (103), and (2-12) crystals, which suggests that the novel sandblasting surface modification process did not change the phase composition of the titanium alloy.

The detection results from our optical contact angle measuring instrument are shown in Figures 4C, D. The contact angle of the surface of the smooth titanium alloy group was 85.26 ± 2.13°, and that of the sandblasted titanium alloy group was 62.03 ± 1.12°. The contact angle of the sandblasted titanium alloy group was evidently smaller than that of the smooth titanium alloy group, and indeed we found the difference between the two groups to be statistically significant (*p* < 0.05) as shown in Figure 4E. Because the contact angle of a material surface is inversely proportional to its hydrophilicity, we may conclude that the hydrophilicity of the surfaces from the sandblasted titanium alloy group is obviously better than those of the smooth titanium alloy group.

### 3.3 *In vitro* experimentation

#### 3.3.1 Cell identification results

**FIGURE 5:**
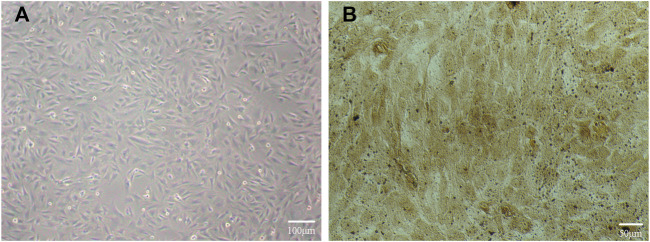
**(A)** Primary cultured osteoblasts in the field of vision under an inverted microscope. **(B)** Primary cultured osteoblasts identification results in the field of vision under an inverted microscope at 200 times magnification.

After 48 h of culture, we found a large number of osteoblasts in the field of vision under an inverted microscope, with irregular polygonal and spindle shapes, abundant cytoplasm, secreted granules, and oval nuclei, as shown in Figure 5A. After we applied the modified calcium-cobalt method, we then observed the cells in the field of vision under an inverted microscope at 200 times magnification, and saw dark-brown deposits in the cell cytoplasm, which were cobalt sulfide particles, as shown in Figure 5B. The above modified calcium-cobalt method is specific to the identification of osteoblasts. Furthermore, according to the results of modified calcium-cobalt method, the primary cultured cells were osteoblasts.

#### 3.3.2 Cell proliferation

**FIGURE 6:**
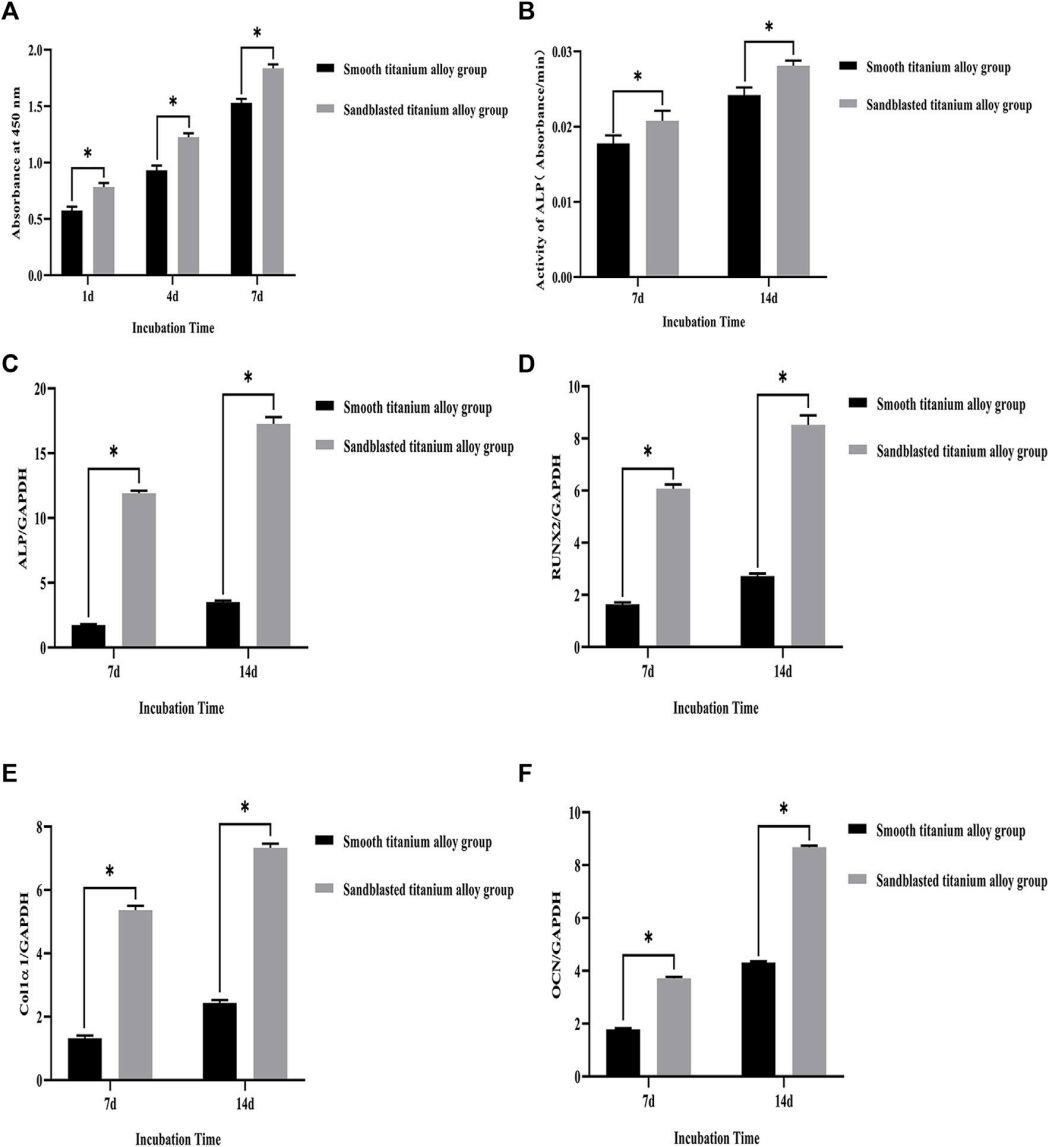
**(A)** CCK-8 test results of the smooth titanium alloy group and sandblasted titanium alloy group at the three incubation time points of 1d, 4d, and 7d. **(B)** The results of ALP activity detection at the two incubation time points of 7d and 14d. The mRNA relative expression levels of the four osteogenesis-related genes at the two incubation time points of 7d and 14d. **(C)** ALP; **(D)** RUNX2; **(E)** Col1α1; **(F)** OCN; GAPDH as the house-keeping gene. ^*^ *p* < 0.05.

Our CCK-8 test results showed that the proliferation of osteoblasts on the surface of the two groups of titanium alloy specimens showed increased with extended incubation time, as shown in Figure 6A. Moreover, the results showed that the sandblasted titanium alloy group showed no cytotoxicity after extended incubation time. In addition, compared to the smooth titanium alloy group, the amount of osteoblast proliferation was higher in the sandblasted titanium alloy group (*p* < 0.05), which further suggests that the titanium alloy implants with bioactive sandblasted surfaces can promote osteoblast proliferation.

#### 3.3.3 ALP activity

The results of ALP activity detection are shown in Figure 6B., where we can see that the ALP activity increased with extended incubation time after osteoblasts were co-cultured with the two groups of titanium alloy specimens. On day 7 of the co-culture, the ALP activity of the sandblasted titanium alloy group was higher than that of the smooth titanium alloy group (*p* < 0.05), and on day 14 of the co-culture, the ALP activity of the sandblasted titanium alloy group was still higher than that of the smooth titanium alloy group (*p* < 0.05). These results suggest that the ALP activity of osteoblasts can be significantly increased by titanium alloy implants with bioactive sandblasted surfaces.

#### 3.3.4 Expression of osteogenesis-related genes

The mRNA relative expression levels of the four osteogenesis-related genes at the two incubation time points of 7 days and 14 days are shown in Figures 6C–F. The expression levels of osteogenesis-related genes on the bioactive surfaces of the sandblasted titanium alloy group were higher than those on the surface of the smooth titanium alloy group (*p* < 0.05), suggesting that titanium alloy implants with bioactive sandblasted surfaces can up-regulate the expression of osteogenesis-related genes. Specifically, the ALP gene experienced the largest up-regulation among the four osteogenesis-related genes.

#### 3.3.5 Type I collagen secretion

**FIGURE 7:**
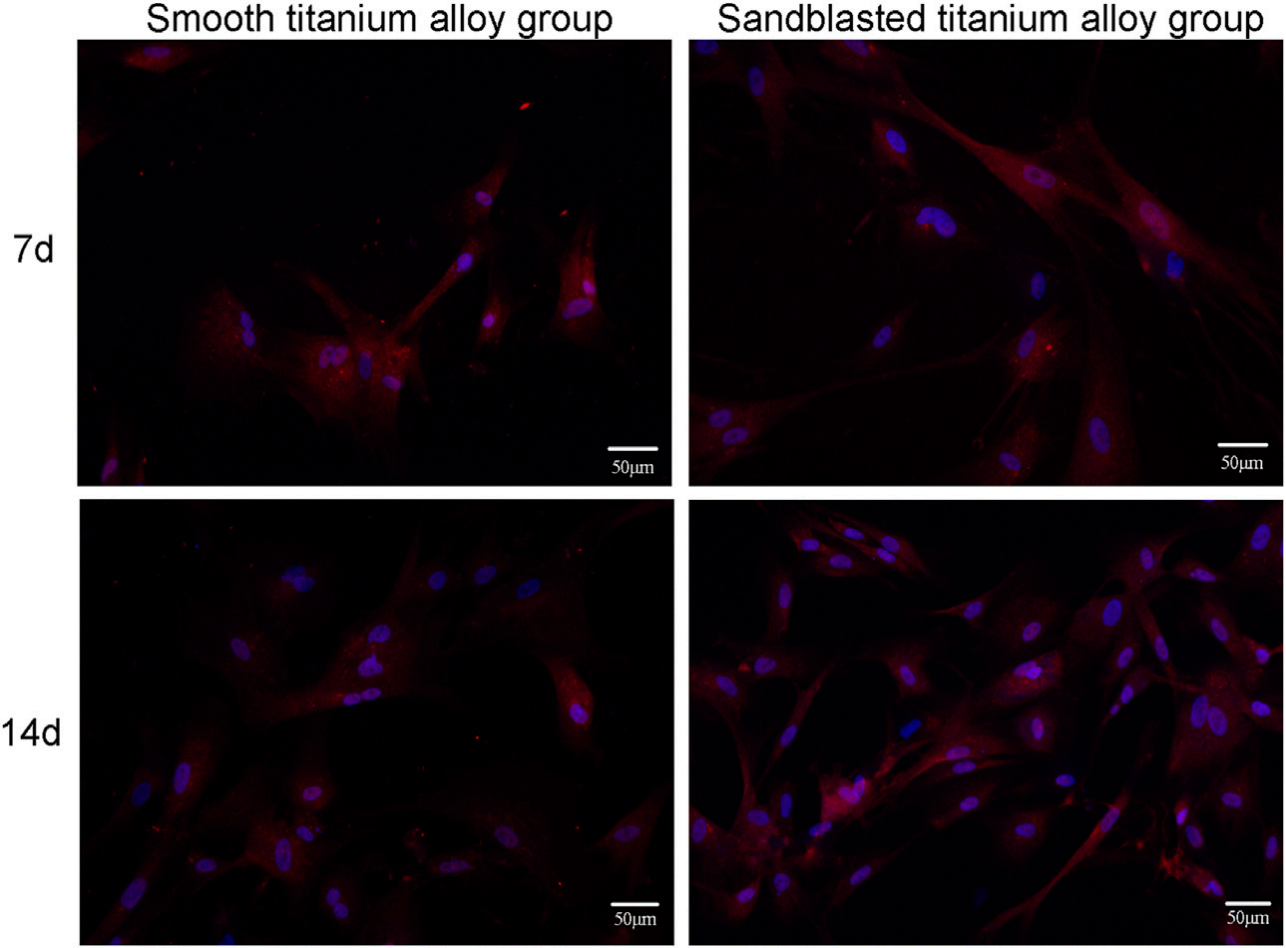
The results of immunocytochemical staining of type I collagen secretion at the two incubation time points of 7d and 14d.

Figure 7. shows the results of our immunocytochemical staining. On day 7 of the co-culture, type I collagen secreted by osteoblasts was distributed on the surface of the two groups of titanium alloy specimens. In addition, the secretion levels of type I collagen from osteoblasts on the surface of two groups of titanium alloy specimens was quite similar. However, on the 14th day of the co-culture, the level of type I collagen secretion from osteoblasts on the bioactive surfaces of the sandblasted titanium alloy group was superior to those on the surfaces of the smooth titanium alloy group, and this result suggests that titanium alloy implants with bioactive sandblasted surfaces can promote the type I collagen secretion in osteoblasts.

#### 3.3.6 Cell growth on the specimen surface

**FIGURE 8:**
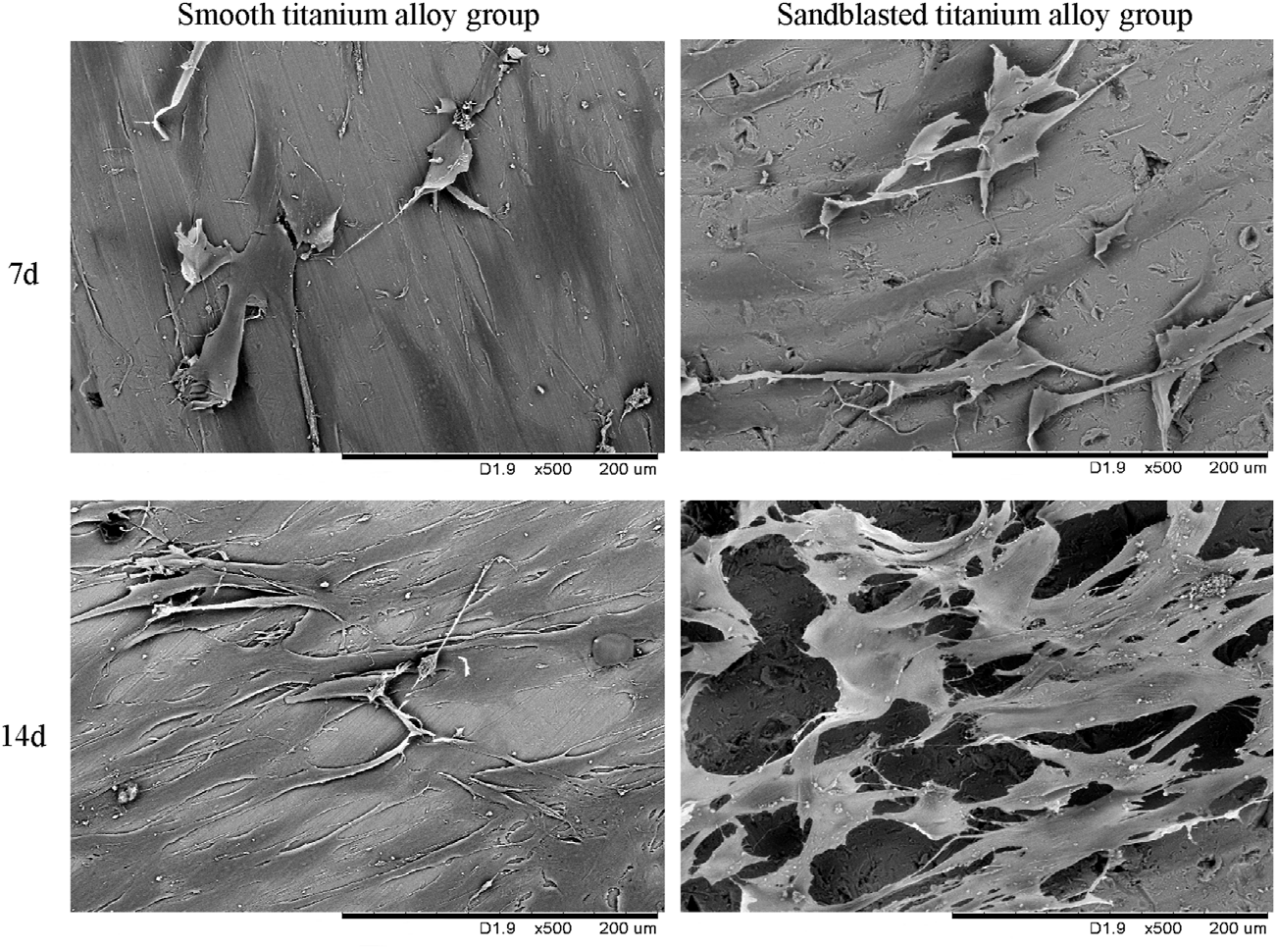
The cell growth detection results under SEM at the two incubation time points of 7d and 14d.

We display the cell growth detection results under SEM with 500 power magnification in Figure 8. In Figure 8, we can see that the osteoblasts showed dendrite-like growth and signs of mineralization on the surface of the two groups of titanium alloy specimens at the 7th day of the co-culture, and the number of osteoblasts with dendritic growth on the surfaces of the sandblasted titanium alloy group was slightly more than that on the surfaces of the smooth titanium alloy group. The osteoblast growth in the sandblasted titanium alloy group was also slightly better than that of the smooth titanium alloy group.

On the 14th day of culture (Figure 8), we observed more osteoblasts on the surfaces of the two groups, and the osteoblasts were in contact with each other, indicating mineralization. However, the osteoblasts in the sandblasted titanium alloy group grew densely and joined together in a single sheet, while the osteoblasts in the smooth titanium alloy group grew sparsely and joined together into a network. Hence, the osteoblast growth of the sandblasted titanium alloy group was better than that of the smooth titanium alloy group. These results suggest that titanium alloy implants with bioactive sandblasted surfaces can better promote the adhesion, proliferation, and mineralization of osteoblasts on the implant surface than smooth titanium alloy implants.

### 3.4 *In vivo* experimentation

#### 3.4.1 Animal tissue toxicity assay

**FIGURE 9:**
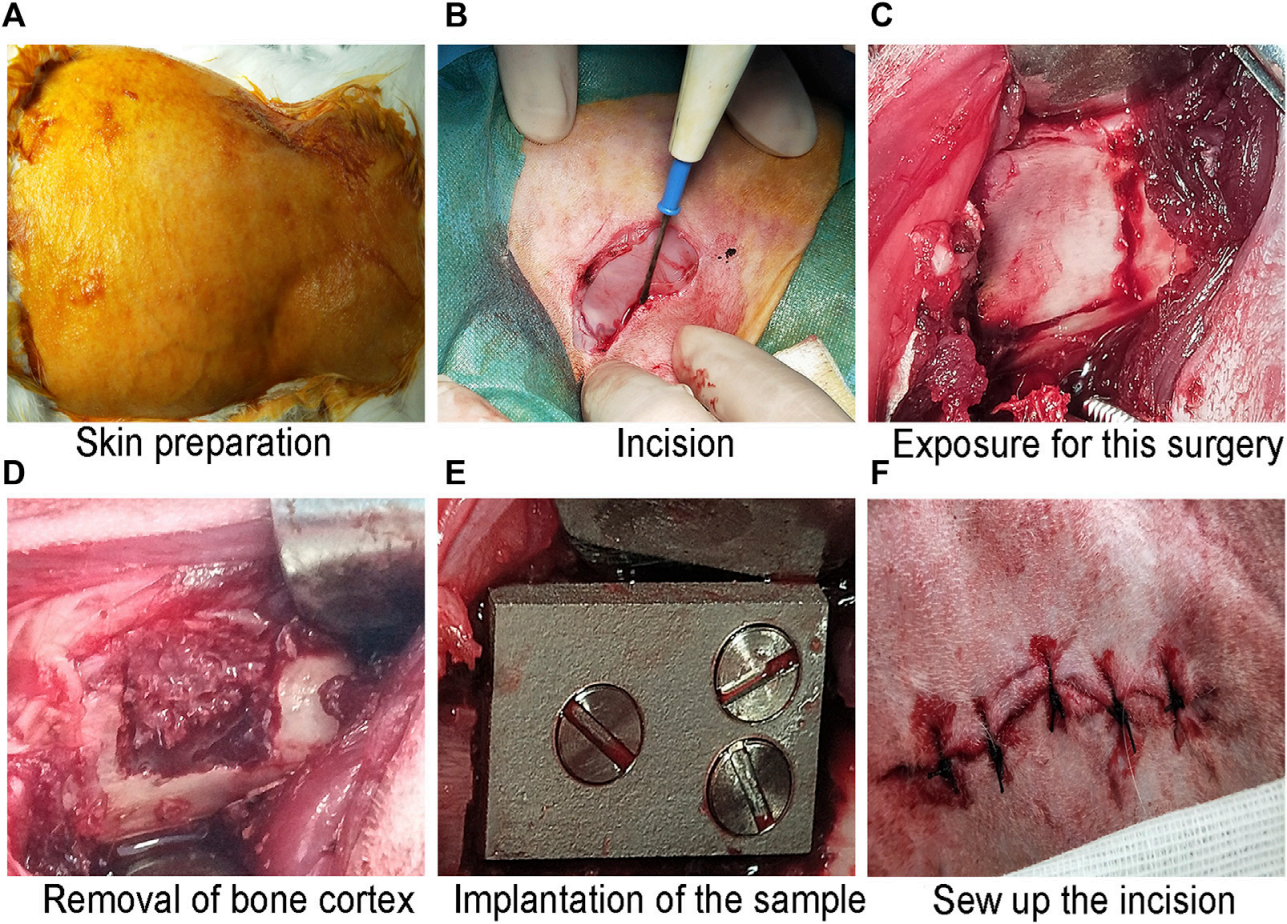
The process of animal model preparation: **(A)** Skin preparation; **(B)** Incision; **(C)** Exposure for this surgery; **(D)** Removal of bone cortex; **(E)** Implantation of the sample; and **(F)** Sew up the incision.

**FIGURE 10:**
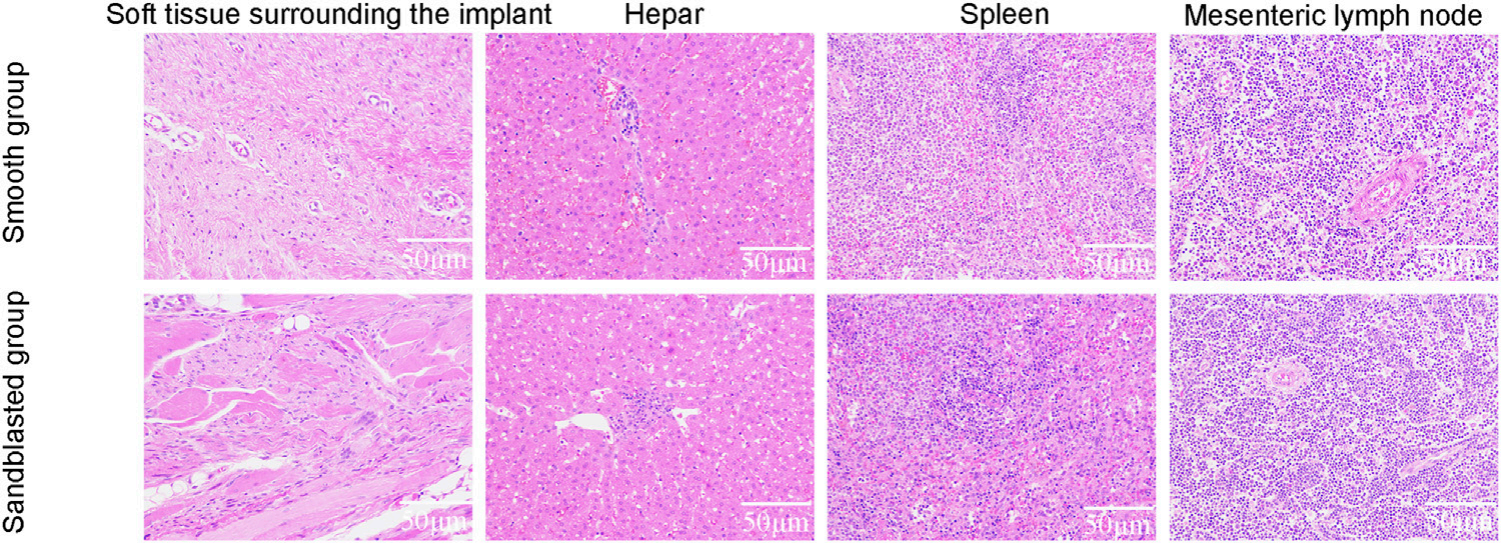
HE staining results.

The process of animal model preparation is shown in Figure 9. Our HE staining results are shown in Figure 10. Here, compared with the smooth titanium alloy group, the adjacent soft tissues of the sandblasted titanium alloy group were normal, and we found no yellow deposition of iron containing blood, no metal particles, and no inflammatory reaction. Furthermore, the morphology of liver, spleen, and mesenteric lymph nodes was normal and free from metal particles or inflammatory reaction. Therefore, we conclude that the titanium alloy implants with bioactive sandblasted surfaces had no toxic effects on tissue.

#### 3.4.2 Micro-CT detection of osseointegration

**FIGURE 11:**
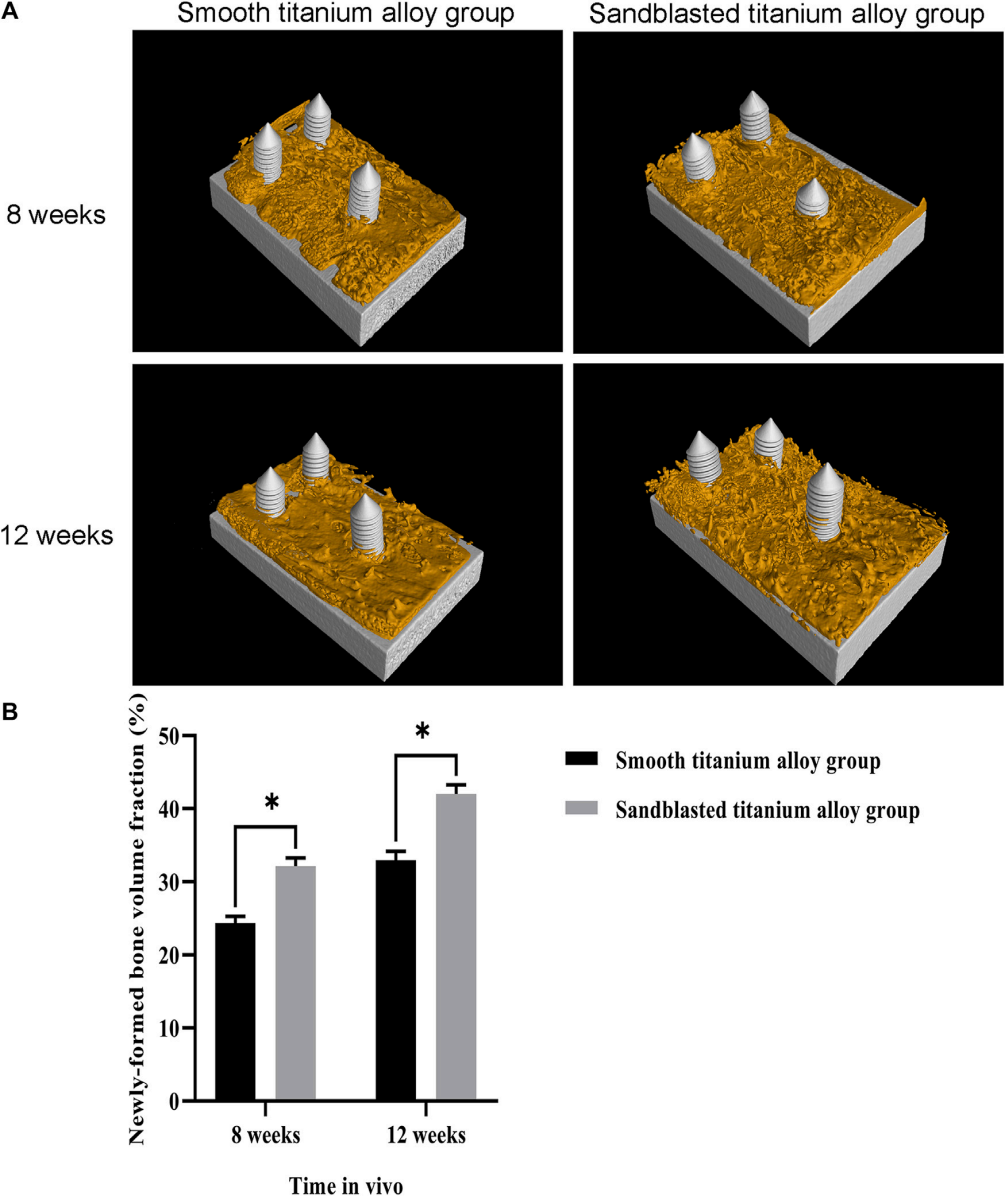
Micro-CT detection of osseointegration **(A)** The results of Micro-CT detection of osseointegration at the two time points of 8 weeks and 12 weeks; **(B)** Analysis of newly-formed bone volume fraction at the two time points of 8 weeks and 12 weeks.

The three-dimensional reconstruction results of the two groups of titanium alloy implants and the surrounding bone tissue by micro-CT are shown in Figure 11A. Here we can see that newly-formed trabecular bone was densely attached to the surface of the two groups of titanium alloy implants, and the yellow area represents the newly-formed trabecular bone within 1 mm of the titanium alloy implant surface. The newly-formed bone volume fraction (newly-formed bone volume/total volume×100%) within 1 mm on the surface of titanium alloy implant was also analyzed, and these results are shown in Figure 11B. At the eighth week, this newly-formed bone volume fraction was 24.34 ± 0.91% in the smooth titanium alloy group and 32.15 ± 1.12% in the sandblasted titanium alloy group. At week 12, it was 32.96 ± 1.23% in the smooth titanium alloy group and 42.04 ± 1.24% in the sandblasted titanium alloy group. Unsurprisingly we found that the newly-formed bone volume fraction in the sandblasted titanium alloy group at the 8th and 12th weeks was statistically higher than that in the smooth titanium alloy group (*p* < 0.05). These results suggest that titanium alloy implants with bioactive sandblasted surfaces can induce more newly-formed trabecular bone formation than smooth titanium alloy implants.

#### 3.4.3 Hard tissue section detection of osseointegration

**FIGURE 12:**
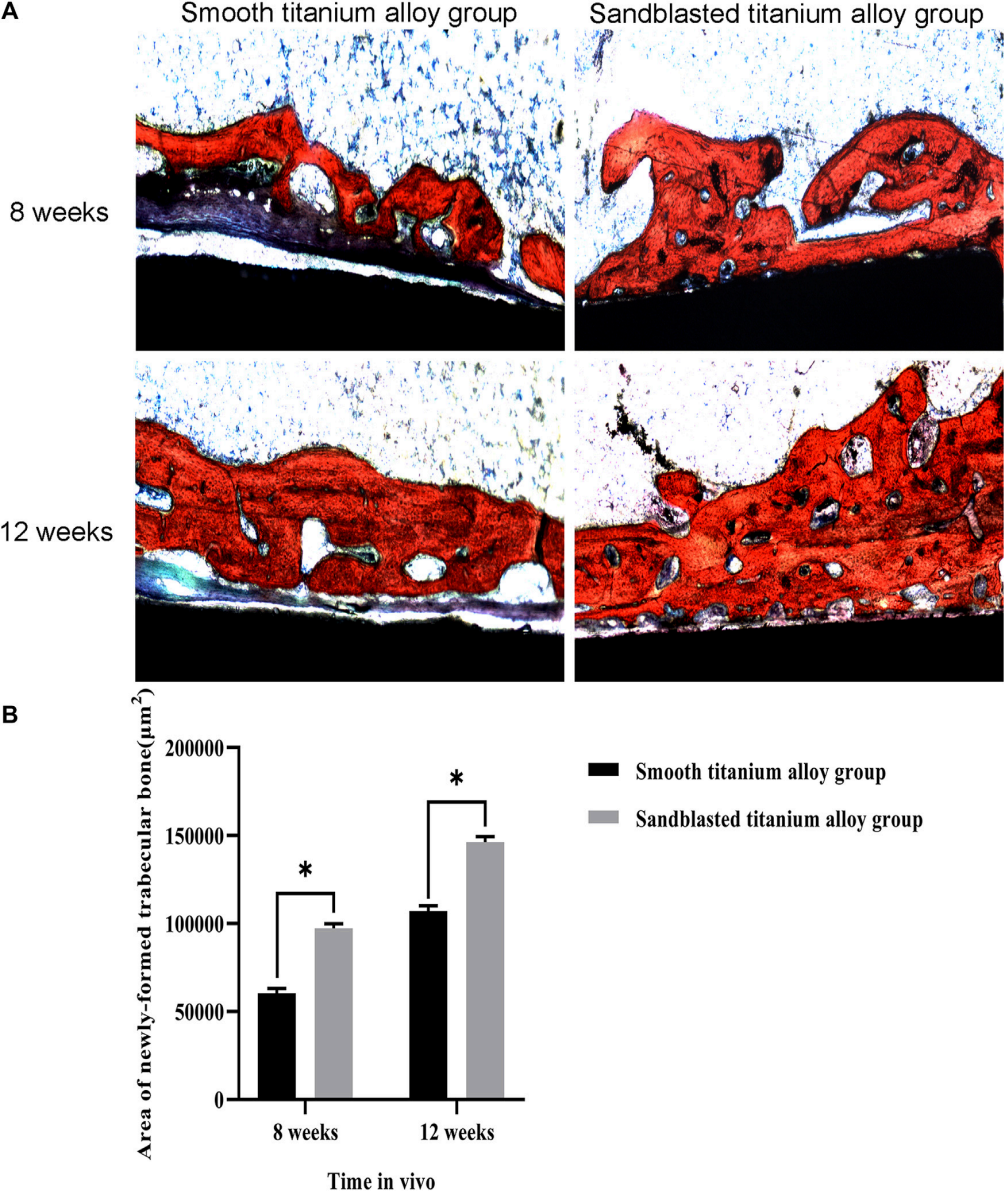
Hard tissue section detection of osseointegration **(A)** The results of hard tissue section detection of osseointegration at the two time points of 8 weeks and 12 weeks; **(B)** Analysis of area of newly-formed trabecular bone at the two time points of 8 weeks and 12 weeks. ^*^ *p* < 0.05.

The observation results of hard tissue section and Van Gieson stain are shown in Figure 12A. In this figure, the black and red sections represent the titanium alloy implant sections and stained newly-formed trabecular bone, respectively. The calculation results of the area of newly-formed trabecular bone are shown in Figure 12B. At the eighth week, the area of newly-formed trabecular bone was 60361 ± 2702 μm^2^ in the smooth titanium alloy group and 97238 ± 2727 μm^2^ in the sandblasted titanium alloy group, and at the 12th week, these areas were 107027 ± 2973 μm^2^ and 146260 ± 3145 μm^2^, respectively. As with the newly-formed bone volume fraction, we once again found that at the 8th and 12th weeks area of newly-formed trabecular bone in the sandblasted titanium alloy group was statistically larger than that in the smooth titanium alloy group (*p* < 0.05), and these results similarly suggest that titanium alloy implants with bioactive sandblasted surfaces can induce more newly-formed trabecular bone formation than smooth titanium alloy implants.

## 4 Discussion

The question of how to improve osseointegration between the material and bone interface is a long-standing problem in modern material science and tissue engineering. Most material studies have focused on the formation of the tissue-implant interface and believe that the tissue-implant interface should remain stable under physiological load conditions. That is, the research framework is that bone tissue can adhere to and grow well on the material surface under these conditions (Weber and Fiorellini, 1992; Parithimarkalaignan and Padmanabhan, 2013; Areid et al., 2021). Due to their inherent biological inertia and stress masking, however, current smooth Ti6Al4V titanium alloy commonly used in clinic have suboptimal osseointegration properties, making it difficult to form good osseointegration with the bone interface (Qin et al., 2018; Wang et al., 2018; Wang et al., 2022). By changing the physicochemical properties and morphology characteristics of titanium alloy material surfaces, we may be able to overcome these shortcomings. In this study, we treated Ti6Al4V with a novel sandblasting surface modification process to form the novel titanium alloy material with bioactive sandblasted surface by changing the surface’s physicochemical properties and morphology characteristics.

After our novel treatment, the composition and proportion of the surface elements of our titanium alloy changed. Compared to smooth titanium alloy, the surface morphology of titanium alloy with bioactive sandblasted surfaces was more complex, with irregularly distributed stacked tile-like bumps on the surface under scanning electron microscopy. This increased the surface area of the titanium alloy and thus increased its contact area with bone tissue. In addition, the AFM results showed an increase in the roughness of the surface of the treated titanium alloy material. The hydrophilicity of the treated titanium alloy materials was also improved, and this may be related to the changes in surface element composition.

Our *in vitro* experiments showed that titanium alloy materials with bioactive sandblasted surfaces produced no signs of cytotoxicity, and the osteoblasts on the surfaces of these materials were more proliferative than those on smooth surface titanium alloy materials as well. We speculate that this is because the novel sandblasting surface modification process changed the chemical composition and morphology characteristics of the titanium alloy material surface.

In this study, we introduced Fe and Si elements to the material surface to change the original elemental composition and proportions of the material surface. The results of our study indicate that changing the elemental composition and proportions of the material surface and introducing Fe and Si elements contributed to the osseointegration. The results of the study by Kopova et al. (Kopova et al., 2016) showed that higher cell population densities and higher collagen yields were obtained from primary human osteoblasts cultured on Ti–35Nb–7Zr–6Ta containing 0.5Si+ 2Fe (wt.%) for 21 days compared to cells cultured on standard Ti-6Al-4V alloy. The results of the other two studies showed that the elements Fe and Si contribute to osseointegration (Mohammadi et al., 2012; Li et al., 2019). The results of our study are similar to those of the three studies mentioned above, which concluded that the introduction of the elements Fe and Si played a role in promoting osseointegration.

Surface morphology with irregularly distributed stacked tile-like bumps, larger surface contact areas, and better hydrophilicity also seem to play an important role in the result. Boyan et al. (Boyan et al., 2017) found that the adhesion of osteoblasts was closely related to the surface morphology and hydrophilicity of implant materials. Similarly, by studying the relationship between the morphology of implant material surfaces and cell proliferation, Brunot et al. (Brunot et al., 2008) found that the complexity of the surface morphology of the material appeared to be positively correlated with cell proliferation. This seems to be related to the fact that the complex surface morphology changes the original roughness and contact area of the material. Our experimental results show that a moderate increase in roughness contributes to osteoblast adhesion and proliferation. In addition, our experimental results suggest that the increased contact area of the material surface offers the possibility for more osteoblasts to adhere and proliferate on its surface. A moderate increase in the surface roughness of the material facilitates osteoblast adhesion, thereby promoting osteoblast proliferation and mineralization (Feng et al., 2003; Price et al., 2004). However, other unexpected factors such as bacterial colonization may occur along with an increase in the surface roughness of the material, as the rough surface seems to facilitate bacterial colonization. The results of one study suggest that the doped surface protects adherent osteoblasts from bacterial colonization and prevents infection prior to osteoblast colonization (Cochis et al., 2020). The relationship between biomaterial surface roughness and the risk of developing infection after biomaterial implantation will be one of our future research directions.

Previous studies have found that cell proliferation increases with an increase in the hydrophilicity of material surfaces as well (Chen et al., 2021; Yu et al., 2021). The surface contact area of material affects the adhesion and proliferation of cells on its surface, and a larger surface contact area is therefore more conducive to the adhesion and proliferation of cells (Yu et al., 2020). The titanium alloy material with bioactive sandblasted surface in our study had the surface morphology with irregularly distributed stacked tile-like bumps, a larger surface contact area, and better hydrophilicity, which are all essential for improving osseointegration between the material and bone interface. The titanium alloy materials with bioactive sandblasted surfaces indeed showed good bioactivity and osteoinductivity in our *in vitro* experiments.

Going further, our *in vivo* experiments showed that the titanium alloy materials with bioactive sandblasted surfaces exhibited no tissue toxicity. Compared with smooth titanium alloy materials, the titanium alloy materials with bioactive sandblasted surfaces induced more newly-formed trabecular bone formation as well. From the analysis of the material itself, the surfaces of the sandblasted titanium alloy materials showed dense and irregularly distributed stacked tile-like bumps, which increased the diversity of surface morphology and was conducive to osseointegration between the material and bone interface. Previous studies have shown that the complex surface morphology of materials is more conducive to the growth of newly-formed trabecular bone compared with smooth material surfaces, because osteoblasts are more sensitive to the complex surface morphology of materials and can better promote the expression of osteogenic genes to regulate the adhesion growth and mineralization of osteoblasts (Hasegawa et al., 2020).

Additionally, our novel sandblasting surface modification process not only changed the surface morphology of the original titanium alloy material, but also increased the surface contact area of the material, and this provided more space for the proliferation and growth of newly-formed trabecular bone. Finally, our novel sandblasting surface modification process also increased the hydrophilicity of the original titanium alloy material surface, which was conducive to osseointegration between the material and bone interface. Osteoblasts are more likely to adhere to and grow on surfaces with good hydrophilic properties (Lotz et al., 2017). In addition, better hydrophilicity of the material surface is conducive to regulating the proteins on the cell membrane and promoting the interaction between cells and the material surface (Sela et al., 2007; Wall et al., 2009; Park et al., 2010). Previous studies have shown that compared to material surfaces with poor hydrophilicity, material surfaces with good hydrophilicity are better at promoting cell adhesion, proliferation, and differentiation, as well as gene expression (Jimbo et al., 2008; Shibata et al., 2010; Li et al., 2012). Finally, the titanium alloy materials with bioactive sandblasted surfaces in our study showed good bioactivity and osteoinductivity *in vivo*, which was consistent with the results of our *in vitro* experiments.

## 5 Conclusion

In this study, we treated Ti6Al4V with a novel sandblasting surface modification process in order to manufacture titanium alloy materials with bioactive sandblasted surfaces. Through *in vitro* and *in vivo* experiments and mutual validation, we found that compared with the current smooth Ti6Al4V titanium alloy materials commonly used in clinic, our titanium alloy materials with bioactive sandblasted surfaces displayed satisfactory bioactivity and osteoinductivity and exhibit good osseointegration properties, resulting in improved osseointegration between the material and bone interface. This work lays a foundation for subsequent clinical application research into titanium alloy materials with bioactive sandblasted surfaces.

## Data Availability

The raw data supporting the conclusion of this article will be made available by the authors, without undue reservation.
